# Investigating the Six-Month Incidence Rate of Burn Disease in Children in Greece

**DOI:** 10.7759/cureus.11192

**Published:** 2020-10-27

**Authors:** Ilias Tsiampouris, Maria Charcharidou, Evangelos Dousis, Niki Oikonomidi, Panagiota Makrygianni, Georgios Vasilopoulos, Ourania Castana, Ioannis Koutelekos

**Affiliations:** 1 Nursing, University of West Attica, Athens, GRC; 2 Nursing, General Hospital of Athens "Georgios Gennimatas", Athens, GRC; 3 Intensive Care Unit, General Oncology Hospital of Kifissia, Athens, GRC; 4 Plastic and Reconstructive Surgery, Evangelismos General Hospital, Athens, GRC

**Keywords:** burns, children, incidence, injuries, burn disease

## Abstract

Introduction

Burns in children are painful, can be fatal, and involve a significant risk of complications, along with physical and psychological consequences. This study aimed to investigate the incidence of burns in children, for six months, and the most common causative factors, along with the existing correlations between demographic data and the characteristics of burn injuries.

Methods

The study was descriptive and prospective, and the sample consisted of minors up to 14 years old with burns in any areas of the body. The research was carried out in the Attica pediatric hospitals’ selected departments for six months (from July to December 2018). Sources for completing the created database were the patients, their guardians, and their medical-nursing documentation and records.

Results

The cumulative six-month incidence rate of childhood burn disease was 4.9%. The most affected age group appeared to be younger than two years (60%), while liquid heat appeared to be the primary form of the burn factor (76%). The average duration of hospitalization for children with a deep partial-thickness to a total-thickness burn degree was 16.5 days. The correlations that emerged related to the extent of the burn were directly related to the accident’s site, and patients with an increased likelihood of future additional surgeries had an increased mean total body surface area that was burned.

Conclusion

Continuous surveillance and removal of hazardous materials from the home environment is of utmost need. Early education/understanding of correct behaviors and proper attention to outdoor activities or excursions can significantly reduce burns. Training courses on burn prevention for parents are needed, as the best form of treatment is prevention.

## Introduction

Burn disease is a serious health condition that can affect multiple systems of the body simultaneously and may lead to acute death and significant mortality [1]. Above all else, it creates a remarkable impact on the patient’s health and the finances of the patients and their families. This makes it challenging to address, and the treatment outcome is critical, not only for adult patients but also for pediatric patients. Burns are among the most common accidents that can occur to children, affecting a fairly large proportion of minors worldwide [2].

In the international literature, studies researching the incidence of burns in children are insufficient [3]. In Greece, burns in minors account for a relatively small but significant number of cases but without proven rates that have been recorded in a common database. Because there is a lack of such studies in Greece, the present study will contribute to further recording and understanding and set goals for the prevention of burn injuries in children, thus demonstrating its importance and need.

## Materials and methods

Research design

The present study was descriptive-prospective, and the sample consisted of minors up to 14 years old with burns in any bodily areas. The research was conducted over six months (from July to December 2018) in selected surgical departments or intensive care unit (ICU) departments of the Attica pediatric hospitals “Athens General Children’s Hospital Panagiotis and Aglaia Kyriakou” and “Athens General Children’s Hospital Agia Sophia.” The demographic-socioeconomic data for the children with burns, the causes and conditions of the burn injuries, the data that determined the severity of burns, and the disease’s outcome were recorded. Finally, an attempt was made to investigate the association between burns’ severity and the demographic and socioeconomic data for the patients.

Data collection process

The research was carried out after the study’s permits were obtained from the pediatric hospitals of Attica. The study start date was July 1, 2018, while the expiration date was December 31, 2018. Daily, over six months, new admissions of burn patients to the pediatric hospitals of Attica’s specialized departments that complied with the study’s criteria were eligible to participate. The patient’s parents or guardians were informed, and then admission to the study proceeded once written consent was received. At the same time, the total number of patients in these specialized departments was recorded daily, and their respective data were entered into the database once informed consent was obtained. Finally, according to the medical record, the date of the patients’ departure, and the outcome of the disease were recorded.

Database

A database was created to collect the following data: demographic-socioeconomic data for children with burn injuries, causes, and circumstances leading to the accident, data identifying the severity of burns, and patient outcome. Resources for filling in the database were patients and their caregivers, medical-nursing documentation forms, and medical records. To assess the severity of the burn, the Lund and Browder map was used to estimate the extent of the burn injuries, and the classification of the depth of the burn was based on clinical evaluation from the medical records [4].

The Lund and Browder map is a useful burn management tool for assessing the total body surface area (TBSA) affected by a burn. It was devised by Charles Lund, a surgeon at Boston City Hospital, and Dr. Newton Browder and was based on their experience in treating over 300 attendees who were injured in a fire at the Cocoanut Grove nightclub in Boston on November 28, 1942. Contrary to Wallace’s rule of nines, the Lund and Browder map takes into account the age of the person, and thus, the percentage of the TBSA for the head is lowered, and the percentage of the TBSA for the feet is increased as a child grows, making it more useful for burns in pediatric patients [4].

Sample of the study

The study sample consisted of patients up to age 14 with any burn injury type, from partial thickness to full thickness, anywhere on their body. The sample collection was carried out in Attica’s pediatric hospitals, specifically, in both ICU departments for children at the hospitals, A and B Surgical Department of the “Aglaia Kyriakou” Hospital and the Plastic Surgery Department of the “Agia Sofia” Hospital.

The patient was included in the study if he or she met the study’s inclusion criteria, which were as follows: (i) boys and girls younger than 14 years old, (ii) patients with burn disease, and (iii) patients with guardians who were able to understand the purpose and process of the study and signed the informed consent form. Patients and their guardians who did not meet the inclusion criteria were excluded from the study.

Over six months, 513 children were admitted to the selected hospitals, 36 of whom had burn injuries. Of the 36 cases, 11 could not be included in the study, as they did not meet the entry criteria. In particular, all 11 of these cases were dismissed because of their burn injuries’ insignificant condition.

Ethics

The current study was accepted by the Scientific Committees of both hospitals (with the decision 221/26.04.2018 for Panagiotis and Aglaia Kyriakou Hospital, and 13005/24-05-2018 for Agia Sophia Hospital) and was conducted under the principle of data protection and confidentiality. The ethical principles, as set out in the Helsinki Declaration, were followed.

Corresponding authorization was also granted by the appropriate committee of the University of Western Attica, formerly known as the Technological Educational Institute of Athens, under the supervision of the Postgraduate Program “Wound Care and Treatment.”

Statistical analysis

Mean, standard deviation, median, and interquartile ranges were used to describe the quantitative variables. Absolute (N) and relative (%) frequencies were used to describe the qualitative variables. Fisher’s exact test was used to compare the proportions. Student’s t-test was used to compare quantitative variables between the two groups. To compare quantitative variables between more than two groups, the parametric analysis of variance was used. Significance levels were two-sided, and statistical significance was set at 0.05. IBM SPSS Statistics for Windows, Version 22.0 (IBM Corp., Armonk, NY) was used for statistical analysis.

## Results

Descriptive results

During the six-month study period (i.e., July through December 2018), 513 children were admitted to the selected hospitals; out of them 25, with burn injuries, were selected for the study. The six-month incidence rate or cumulative incidence of childhood burn disease was 4.9%. Boys accounted for 76% of the children. The age of children with burn injuries ranged from 11 months to 14 years, with a mean of 3.6 years (standard deviation [SD], 4.0 years). Seventy-two percent of the children were Caucasian and were also of Greek nationality. Fifty-six percent of children lived in Attica, and the remaining 44% lived in a remote/rural region. Sixty percent of the children attended nursery school.

Nevertheless, 84% of the children were members of a nuclear family. None of the children had comorbidity, mental disorders, motor problems, or mental illness. The demographic data for children with burn injuries and their backgrounds are presented in Table 1.

**Table 1:** Patient demographics

Demographic data		Ν	%
Sex	Male	19	76.0
	Female	6	24.0
Age (years)	<2 years	15	60.0
	≥2 years	10	40.0
Age (years), mean (SD); median (interquartile range)		3.6 (4.0)	1.6 (1-5.5)
Race	Caucasian	18	72.0
	Colored	6	24.0
	Other	1	4.0
Citizenship	Greek	18	72.0
	Foreigner	7	28.0
Place of residence	Attica	14	56.0
	Remote region	11	44.0
Patient’s educational level	Nursery school	15	60.0
	Day nursery school	1	4.0
	Pre-kindergarten	1	4.0
	Kindergarten	4	16.0
	Primary school	2	8.0
	High school	2	8.0
Patient’s marital status	Nuclear family	21	84.0
	Extended family	1	4.0
	Single-parent family	3	12.0
Existence of co-morbidity		0	0.0
Existence of mental disorders		0	0.0
Existence of motor problems		0	0.0
Existence of mental disorders		0	0.0

The mean hospital stay was 16.5 days (SD 12.2 days). The majority of children (72%) were transferred to the hospital by private means of transportation. Information on the children’s hospitalization is provided in Table 2.

**Table 2:** Patient’s transferring and hospitalization records

Transferring and hospitalization records		Ν	%
Duration of hospitalization (days), mean (SD); median (interquartile range)		16.5 (12.2)	14.5 (8-21)
Mean of transport	Ambulance	7	28.0
	Private mean of transport	18	72.0

All children had thermal burn injuries. Eighty-eight percent of children had a mixed depth of burn injuries, and the same percentage had multiple burned areas. Only one child had an inhalant burn injury. The mean area of burn injury was 16.9% (SD 8.8%). Information on the children’s burn injuries is provided in Table 3.

**Table 3:** Patient’s burn injury data

Burn injury data		Ν	%
Type of burn injury	Thermal	25	100.0
Depth of burn injury	Deep partial thickness	3	12.0
	Mixed depth	22	88.0
Multiple burned areas	No	3	12.0
	Yes	22	88.0
Existence of inhalation burn injuries	No	24	96.0
	Yes	1	4.0
Mean area of burn injury (%), mean (SD)		16.9 (8.8)	

The most frequent burn injuries were on the trunk’s anterior surface, neck, head, and anterior surface of the right hand with 64.0%, 56.0%, 44.0%, and 40.0%, respectively. Table 4 provides a detailed breakdown of the children’s burn injuries in a descending order.

**Table 4:** Patient’s burn injuries in a descending order

Body area	Ν	%
Anterior trunk surface	16	64.0
Neck	14	56.0
Head	11	44.0
Right hand anterior surface	10	40.0
Left hand anterior surface	8	32.0
Right hand rear surface	6	24.0
Trunk rear surface	5	20.0
Right foot anterior surface	5	20.0
Left foot anterior surface	5	20.0
Buttocks	4	16.0
Left hand rear surface	3	12.0
Right foot rear surface	3	12.0
Left foot rear surface	3	12.0
Right palm outer surface	2	8.0
Right palm inner surface	2	8.0
Perinatal area	2	8.0
Right foot outer surface	2	8.0
Left foot outer surface	2	8.0
Left palm exterior surface	1	4.0
Left palm inner surface	1	4.0
Right insole inner surface	1	4.0
Left insole inner surface	1	4.0

The main cause of burn injuries was wet heat (76.0%). The most common household location where the injury occurred was the kitchen, with a 60.0% rate, and most accidents occurred either at noon or in the morning, with rates of 48.0% and 36.0%, respectively. Table 5 provides information on the accident that caused the burn injuries, while Table 6 provides data on the treatment and the therapy outcome.

**Table 5:** Accident information

Accident information		Ν	%
Cause of burn	Dry heat	4	16.0
	Wet heat	19	76.0
	Hot object	2	8.0
Accident area	Kitchen	15	60.0
	Bathroom	2	8.0
	Living room	5	20.0
	Outdoors	3	12.0
Time during the day the accident occurred	Morning	9	36.0
	Midday	12	48.0
	Afternoon	3	12.0
	Night	1	4.0

**Table 6:** Patient’s treatment and therapy outcome data

Patient's treatment and therapy outcome data		Ν	%
Treatment of burn injuries	Conservative therapy	23	92.0
	Surgical therapy	2	8.0
Skin transplant	No	24	96.0
	Yes	1	4.0
Type of implant	Autograft	1	4.0
	None	24	96.0
Signs of infection	No	25	100.0
	Yes	0	0.0
Possibility of future additional surgeries	No	18	72.0
	Yes	7	28.0
Final outcome	Complete cure	5	20.0
	Discharge with continuous observation	15	60.0
	Discharge with scheduled readmission	2	8.0
	Death	0	0.0
	Patient stayed after completion of investigation	3	12.0

Correlation of burn extent, demographic data for children, and burn injury data

The extent of children’s burn injuries was similar concerning their demographics. Table 7 provides information on the extent of children’s burns according to their demographics.

**Table 7:** Correlation of burn injury extent and demographics data

Patients demographics		Extent of burned body area (%)		p, Student's t-test
		Mean	SD	
Sex	Male	18.4	8.4	0.147
	Female	12.3	9.5	
Age (years)	<2	16.7	7.4	0.851
	≥2	17.4	11.0	
Race	Caucasian	16.0	9.9	0.423
	Black/Other	19.2	5.4	
Citizenship	Greek	16.8	9.2	0.894
	Foreigner	17.3	8.5	
Nuclear type of family	No	20.5	7.5	0.389
	Yes	16.3	9.1	

It is worth mentioning that the burned area was significantly higher in children who experienced an accident outdoors, with an average burned TBSA rate of 29.0%, compared to children who received burns inside their home, with a mean burned TBSA rate of 15.3%. (p = 0.008). Nevertheless, a significantly higher range of burned area was found in children treated with surgery, with an average burned TBSA rate of 30.0% (p = 0.026), as well as those who were likely to have future additional surgeries, with a mean burned TBSA rate of 25.1% (p = 0.002). Table 8 shows the extent of children’s burn injuries according to their burn data.

**Table 8:** Correlation of burned body area extent and burn injury data

Burn injury data		Extent of burned body area (%)		p, Student's t-test
		Mean	SD	
Cause of burn	Dry heat	25.1	11.1	0.119
	Wet heat	15.1	8.0	
	Hot object	17.5	3.5	
Accident area	Home	15.3	7.5	0.008
	Outdoors	29.0	10.1	
Treatment	Conservative treatment	15.8	7.7	0.026
	Surgical treatment	30.0	14.1	
Possibility of future additional surgeries	No	13.7	7.1	0.002
	Yes	25.1	7.8	
Final outcome	Complete cure	14.4	6.6	0.486
	Discharge with continuous observation/discharge with scheduled readmission/patient stayed after completion of investigation	17.6	9.3	

Correlation of outcome, demographic data, and burn injury treatment data

The outcomes of the children, according to the data collected, were not significantly different. Table 9 shows the outcome of the children according to their demographics and the data related to the treatment of burns.

**Table 9:** Correlation of patient’s outcome, demographic, and treatment data

Patient demographics and treatment data		Patient's outcome				p, Fisher's exact test
		Complete cure		Discharge with continuous observation/discharge with scheduled readmission/patient stayed after completion of the investigation		
		N	%	N	%	
Sex	Male	3	15.8	16	84.2	0.562
	Female	2	33.3	4	66.7	
Age (years)	<2	3	20.0	12	80.0	1.000
	≥2	2	20.0	8	80.0	
Race	Caucasian	4	22.2	14	77.8	1.000
	Black/Other	1	14.3	6	85.7	
Citizenship	Greek	4	22.2	14	77.8	1.000
	Foreigner	1	14.3	6	85.7	
Treatment	Conservative treatment	4	17.4	19	82.6	0.367
	Surgical treatment	1	50.0	1	50.0	
Possibility of future additional surgeries	No	5	27.8	13	72.2	0.274
	Yes	0	0.0	7	100.0	

## Discussion

This study’s cumulative incidence was determined to be 4.9% for the study period for children who required hospitalization in pediatric hospitals. Although there are no similar surveys in Greece to compare the results, nor are there similar, relatively recent, European country surveys, similar studies have been conducted in other countries worldwide, and our results appear to be quite close to them. Specifically, in 2016, in a US study, burn incidence for 1998 to 2013 was determined, in which severe thermal burn injuries were included, just as in the present Greek study, at 2.15% [5]. In contrast was a 2017 study for 2000 to 2014 in Brazil, with an incidence at 14.56% that included children under the age of five years who accounted for 24% of imports [6], while for 2016, a similar proportion was also found in the Zuni region of southwestern China, with an impact of 12.7% [7]. It should be noted that the burn incidence of the present study was equal (4.9%) to that of the city Riyadh of Saudi Arabia for 2013 [8].

Of the 25 cases in the present study, 76% were boys (n = 19), and 24% were girls (n = 6). Similar results have been obtained in various studies worldwide, such as in one study in Croatia in 2015, with boys accounting for 64.15% and girls accounting for 35.85% [9]. In the United States in 2016, boys accounted for 68%, and girls accounted for 32% [10], and in other countries such as Bangladesh [11], Kenya [12], and Nigeria [13], the highest number of cases was for boys. However, the distribution of nonfatal serious burn injuries by sex varies between countries. In some countries, such as Egypt and India, a higher rate of burn injuries was obtained for girls [14]. Pediatric patients consisted of 72% Caucasian and 24% other races, with rates similar to those reported by the American Burn Association in 2016 [10].

Fifty-six percent of the cases in the current study lived in the urban area of Attica, compared to 44% who lived outside of Attica, which is roughly the same as figures from the United States (54% for urban and 46% for remote/rural areas) [15] and similar to a 2015 Croatian survey, where 67% of cases resided in an urban area, compared to 33% in a rural area [9]. Finally, in 2015, an Australian study for 2008 to 2012 accounted for 21.2% of incidents from rural areas compared to 78.8% of incidents from urban areas [16].

Although 84% of the sample in the current study belonged to a nuclear family, as opposed to 12% who belonged to a single-parent family, and there were no children in the sample who had comorbidities, mental disorders, motor problems, or mental illness, after searching the international bibliography, there was not a single previous study that investigated the correlation of burn injuries and patients’ marital status or the presence of concomitant diseases.

The average length of hospitalization for children with deep partial-thickness to total-thickness burn injuries was 16.5 days. Similar rates appeared in a South Korean study, with a mean hospital stay of seven days [17], and in an Australian study, with a mean hospital stay of three days [18]. In contrast, a similar study in central China showed that pediatric patients required an average length of stay of 52.3 to 40.2 days [19], while in Croatia, the mean hospital stay was 174.5 days [9].

Children under the age of two years represented 60% of the study (n = 15), while children over the age of two represented 40% (n = 10). Although several studies worldwide reported that most burn injuries occurred in children under the age of two, there are studies with similar rates, such as a Beijing study, in which 47.8% were children over the age of two and 52.2% were under the age of two [20], and an Indian study, where it was shown that 50.1% of participants were over two years old and 49.9% were younger than two years [21].

All 25 cases (100%), which met the criteria for entry into our study, were treated for heat burn, unlike worldwide studies where the sample differed due to the presence of an electrical or chemical agent. Only 12% (n = 3) had deep partial-thickness burns, as opposed to the very high 88% (n = 22), with mixed-depth burn injuries (mainly total thickness as well as deep partial-thickness). A literature review found only two studies reporting the rates of tissue damage depth, consisting of a Central China study in 2017, where a lower percentage of total-thickness burn injuries, at 59.3%, and a higher percentage of partial burn injuries, at 40.7%, were shown [19], and an Indian study in 2017 that showed that 49.5% had partial-thickness burn injuries, while 41.9% had mixed-thickness burn injuries [21]. At the same time, a 2016 study in England only reported the incidence of severe thermal burn injuries of full-thickness, without reporting the proportion of children [5].

Eighty-eight percent (n = 22) of the current study cases had multiple burned areas, as opposed to 12% (n = 3), where burn injury occurred on only one part of their body. A study in Morocco found that the proportion of children burned in more than one area was 69.03% [22].

Inhalation burn injury accounted for 4% of the sample in the present study, which corresponds to only one patient. Its incidence is much higher in overseas studies, as the recording time is longer, making the study sample significantly larger.

The mean burned area in this study was 16.9%. In studies abroad, there was a rating of 5% in a Saudi Arabian study [8], 5.5% in a Moroccan study [22], 19.73 ± 15% in an Iraqi study [23], and 37 ± 24% in an Indian study [21]. The three main areas of the body that suffered burn injuries, in a descending order of incidence, are the trunk’s anterior surface with 64%, the neck with 56%, and the head with 44%. In contrast to a study in South Korea the same year, the most frequently injured area of the child’s body, in the descending order, was the upper limbs, lower limbs, and body trunk, with no percentages, reported [17]. However, a study from Saudi Arabia listed face, chest, and knees as the most common sites of burn injuries [8].

The burn accident’s primary cause was 76% due to wet heat, followed by 16% caused by dry heat, and 8% due to contact with hot objects. Wet heat appears to remain first, as shown in a 1996 burn injury study in France with 64.1% and 16.95% for volatile fluids [24]. Similar results were found in studies conducted in Peru in 2002, with wet heat accounting for 74% of the cases [25], Bradford in 2007 (52%) [26], Morocco in 2013 (83.5%) [22], and India in 2017 (48.8%), followed by dry heat (43.4%) [21].

Our survey showed that most burn injuries occurred at home (88%), compared to 12% for outdoor burn injuries. In particular, 60% of accidents occurred in the kitchen, 20% occurred in the living room, and 8% occurred in the bathroom. The 1996 French study agrees once more with the present data, stating that the kitchen is the most dangerous area of the house, with 56.2% occurrence, while the bathroom is in second place with 13.6% [24]. Despite a 10-point difference, a Peruvian study also showed that most accidents occur at home (77.5%), and the most common area where accidents occur was the kitchen (67.8%) [25]. According to a South Korean study, the majority of burns took place indoors, with the home being the most common area (85.8%), and more specifically, the kitchen/dining area comprising 58.8%, living room 23.3%, bedroom 11.4%, and bathroom 5.1% [17]. A sub-Saharan African study showed that almost 89.2% of pediatric cases were injured at home [27], while a study conducted in Riyadh showed that burn injuries caused at home accounted for 35%, compared to 2.7% that occurred outside the home [8]. In contrast, a study in England in 2007 showed that 85% of burn injuries had occurred in nature/the outdoors [26].

Most of the accidents in our study occurred in the morning and midday, at 36% and 48%, respectively. During the afternoon, 12% of the accidents occurred, while in the evening, there was only one incident, with the rate reaching 4%. Only one transcontinental study reported that the two principal hours in which accidents occurred were late morning and afternoon [28].

The leading cause of burn injuries appeared to be due to hot liquids in the kitchen or living room at 72%, including water, beverages, food, or oil. Thereafter, the cause appeared to be the child in 12% of cases, due to a lack of adult supervision in the same room. The third cause was bathing for 8%, and the last was home fires at 8%, due to external or internal factors. To the best of our knowledge, there has been no similar study in the international literature that investigated the accident’s cause and the extent of patient involvement in causation.

Most children (92%) received conservative treatment, while 8% underwent surgical treatment. Only one child (4%) received a transplant, and autografts were used in particular, while 28% of children were candidates for future additional surgeries. In other studies, 35% of patients required a skin graft [24], while another study found that children living in a rural area required a skin graft in 28.3% of cases, as opposed to children who were residents of urban areas, of whom 16.3% required a skin graft [16]. In another study, it was observed that over 10 years from 2003 to 2013, the need for skin grafts decreased from 18% to only 5% [29].

The outcome was that 60% of the patients were discharged with continuous follow-up, while 8% had been discharged with a scheduled reintroduction to perform surgeries. In a study by the Australian and New Zealand Burn Association in 2017, almost twice as many (14%) children were re-admitted within 28 days of the discharge day, with the majority (77%) reported as planned reintroduction [18].

The correlation of the extent of children’s burn injuries and their demographics showed that the extent of burn injuries was similar for each child’s differentiation data, with no noticeable difference. However, the correlation between the extent of burn injuries and the burn injury data was statistically significant. Specifically, the extent of the burn injury was directly related to the accident site, with the mean value of the burned TBSA at-home accidents being 15.3%, compared to 29% for accidents occurring outdoors (p = 0.008). At the same time, the mean value of the extent of the burned area was statistically significant (p = 0.026), as patients with conservative treatment had a mean burned TBSA of 15.8%, compared to patients who underwent surgical treatment, who had 30% of the average burned TBSA.

Finally, there was statistical significance (p = 0.002) among those who had an increased likelihood of future additional surgeries, with a mean burned TBSA of 25.1%, as opposed to those who did not have such a probability, with an average burned TBSA of 13.7%. As far as we are aware, this specific association (burn area-demographic burn-out data) and the concerned data haven't been studied before.

Correlating the outcome with patient demographics and the treatment of burn injuries, pediatric patients’ outcomes did not significantly differ, with the data being shared between differentiation data. To our knowledge, no studies were found to examine this specific association (patient outcome-demographic-burn-out) and present their data.

Study limitations

This study was subject to methodological limitations. The sample size of the study, although small, is a convenience sample and is not representative of the population to yield sufficient statistically significant results. The present study would probably require a longer time to record more cases and observe the sample. The information collected relates to pediatric patients with burn disease treated in the selected hospitals and not to those who came to the emergency departments. Finally, the sample that was recorded concerned only data from the Prefecture of Attica’s pediatric hospitals, specifically, the Athens General Children’s Hospital “Panagiotis and Aglaia Kyriakou” and Athens General Children’s Hospital “Agia Sophia.”

## Conclusions

The acquisition of burns and the severe effects they may have on childhood and their impact on human life have prompted research questions to be initiated. Boys seem to be more prone to burn accidents related to the lively side of their nature. Infants and toddlers seem to be more prone to exploring and discovering the area around them, but unfortunately, they are unaware of the dangers that could lead to an accident. In neither case can special precautionary or preventive measures be taken except to recommend the continuous surveillance and monitoring of children inside and outside the home, to remove hazardous materials that may cause combustion, chemical burns, or electric shocks, as well as informing children early on regarding proper behavior, what is dangerous, what the results can be, and what can cause serious injuries to them.

Most accidents seemed to occur inside the home and mainly in the kitchen, when cooking was being conducted, and while parents were in the same room. Parents should make sure that children do not enter the kitchen during cooking, or when they enter, that their safety is ensured. A parent should never hold a child in his or her arms while they cook because children tend to explore the area around them and can cause an accident. Therefore, it seems training courses on burn prevention for parents are needed, as the best form of treatment is prevention. Lastly, burn accidents caused outdoors appeared to cause more extensive burn injuries at both the surface and depth. Parents and children should pay particular attention when going on nature trips and setting up improvised fireplaces, as severe burns can easily result from such fires.

When considering the higher validity and reliability of the results, it is clear that there is an urgent need to design future studies that will provide a larger sample that is representative of the population and be carried out for a more extended period of time so that they can produce sufficient important statistical results. At the same time, the information should be collected for the entire pediatric population through primary and secondary education (schools) or emergency departments to record more cases, even those that do not require hospitalization. Future studies should compare the results between two towns or at least the incident recordings, go beyond the scope of a region, and be conducted within a geographical department or even the entire country.
